# The Effectiveness of an Online Support Group for Members of the Community with Depression: A Randomised Controlled Trial

**DOI:** 10.1371/journal.pone.0053244

**Published:** 2012-12-28

**Authors:** Kathleen M. Griffiths, Andrew J. Mackinnon, Dimity A. Crisp, Helen Christensen, Kylie Bennett, Louise Farrer

**Affiliations:** 1 Centre for Mental Health Research, The Australian National University, Canberra, Australian Capital Territory, Australia; 2 Biostatistics Unit, Orygen Youth Health Research Centre, University of Melbourne, Melbourne, Victoria, Australia; 3 Centre for Research in Ageing, Health and Wellbeing, The Australian National University, Canberra, Australian Capital Territory, Australia; Linkoping University, Sweden

## Abstract

**Background:**

Internet support groups (ISGs) are popular, particularly among people with depression, but there is little high quality evidence concerning their effectiveness.

**Aim:**

The study aimed to evaluate the efficacy of an ISG for reducing depressive symptoms among community members when used alone and in combination with an automated Internet-based psychotherapy training program.

**Method:**

Volunteers with elevated psychological distress were identified using a community-based screening postal survey. Participants were randomised to one of four 12-week conditions: depression Internet Support Group (ISG), automated depression Internet Training Program (ITP), combination of the two (ITP+ISG), or a control website with delayed access to e-couch at 6 months. Assessments were conducted at baseline, post-intervention, 6 and 12 months.

**Results:**

There was no change in depressive symptoms relative to control after 3 months of exposure to the ISG. However, both the ISG alone and the combined ISG+ITP group showed significantly greater reduction in depressive symptoms at 6 and 12 months follow-up than the control group. The ITP program was effective relative to control at post-intervention but not at 6 months.

**Conclusions:**

ISGs for depression are promising and warrant further empirical investigation.

**Trial Registration:**

Controlled-Trials.com ISRCTN65657330

## Introduction

The Internet is increasingly recognised as a valuable self help resource for people with depression. It has been estimated that as many as 21% of US citizens have accessed online information about depression, anxiety, stress or mental health issues [1]. This high level of mental health information seeking on the Internet is not surprising given the prevalence of mental disorder in the community [2], the fact that depression is the leading cause of years lost to disability globally [3] and the evidence that health information seeking on the Internet is higher among those with stigmatised health conditions [4].

However, Internet usage for depression is not limited to information seeking. Recent systematic reviews have concluded that guided and unguided online psychotherapy applications are effective for preventing and treating depression [5], [6]. Further, the evidence-based UK NICE guidelines for depression recommend that people with mild depression be offered access to computerised cognitive behaviour therapy [7]. Less is known about another emerging forms of online self help: Internet support groups (ISGs) from which members of the public seek information and support from others with a similar condition. An estimated 28% of US Internet users access such groups [8] and there is evidence that ISGs are particularly popular among consumers with depression [9]. However, a systematic review [10] and follow-up searches failed to yield any randomised controlled trials of the effectiveness of ISGs for depression. On the other hand, there is promising evidence of the effectiveness of conventional mutual support, with a recent systematic review of studies of *face*-to-*face* support groups for depression concluding that mutual support was more effective than treatment as usual and that there was no statistically significant difference in the efficacy of support groups and cognitive behaviour therapy [11]. Since ISGs have the potential to increase the accessibility of mutual support there is a need to determine if they, too, are effective. Such groups might also serve a second role. Online psychotherapeutic interventions are often characterised by a high attrition rate [12]. It has been suggested that social support might increase adherence to medical treatments [13]. If this is the case, an online peer-to-peer support group might facilitate adherence to an automated online psychotherapy intervention through mutual encouragement. To date however, the effect of an adjunct support group on intervention adherence and health outcomes has not been examined.

The current study therefore sought to evaluate the efficacy of and adherence to a depression Internet Support Group (ISG), an automated psychoeducational and skills Internet training program (ITP), and the ITP combined with an ISG relative to each other and a plausible Internet Attention Control condition (IAC).

## Methods

The protocol for this trial and supporting CONSORT checklist are available as supporting information; see Checklist S1 and Protocol S1. Since the WellBeing trial protocol has been published in detail elsewhere [14], the current paper contains only a brief description of the methods employed. Participants with elevated levels of depressive symptoms were randomised to receive one of four conditions: the ISG, the ITP, a combination of the ISG and the ITP, or a plausible Internet-based Control intervention. Assessments were undertaken at baseline, at post-intervention (end-point), and at 6- and 12- month follow up. The trial was approved by The Australian National University Human Research Ethics Committee (Protocol 2007/2259) and registered with the Controlled Clinical Trials registry (ISRCTN65657330).

### Participant recruitment and setting

Participants comprised 311 adults aged 18 to 65 years recruited in three waves between August 2008 and May 2009 via a screening survey posted to 70,000 adults randomly selected from the electoral rolls of eight Australian electoral divisions (4 rural, 4 metropolitan) with moderate to high Internet access. To be included in the intervention study, respondents were required to have obtained a Kessler Psychological Distress (K10) [15] score of more than 22, to have home or work access to the Internet and to consent to participating in the study. Those receiving treatment from a mental health professional or CBT or who were participating in a mutual support group or another research project at the lead investigator's research centre at the time of recruitment were excluded. Potential participants were also excluded if they self-reported current or past experience with or diagnosis of psychosis, schizophrenia or bipolar disorder.

Randomisation to one of the four trial conditions was undertaken following verbal consent on the telephone prior to the return of written consent [14]. At this point neither the participant, nor the interviewer nor the project coordinator was aware of the participant's randomisation status. Accordingly, dropout at this stage could not have been influenced by the condition to which the participant was randomised. Those participants who returned informed consent forms were sent a letter advising them of the condition to which they had been randomised together with a user guide to the trial and their intervention. Baseline data were collected thereafter.

### The Interventions

Each of the three interventions and the Control program comprised 12 modules, one module delivered per week over a 12 week period. Once opened, these programs remained accessible to participants during the trial and 12 month follow-up period. For ethical reasons, participants in the control condition were provided with access to the ITP after the 6-month follow-up assessment. The ITP, ISG and IAC conditions employed the e-couch, WellBeing Board and HealthWatch websites respectively. The content of each intervention website is summarised below and described in detail in the WellBeing protocol paper [14].

#### ITP: E-couch (depression stream)

The ITP comprised a research version of the depression stream of e-couch, an automated online psychological intervention application (http://ecouch.anu.edu.au). The intervention consisted of (i) a depression literacy module containing consumer information about the diagnosis, epidemiology and treatment of depression; and (ii) a self help module comprised of online versions of cognitive behaviour therapy; interpersonal therapy; applied relaxation; and physical activity.

#### ISG: WellBeing Board

The ISG was a closed, moderated bulletin board purpose built for the trial. The structure and rules for the board were based on our public depression forum BlueBoard (http://blueboard.anu.edu.au). Each week one or two new forums or topics were introduced to one of the three main WellBeing Board categories: Your WellBeing, Feeling Better, or General [14]. Participants were asked to login to the board at least twice weekly to read new messages posted by other members of the ISG and to contribute at least four posts to the board weekly on the current topic or past week's topics or other issues. The Board rules were displayed at first registration and were available at all times on the Board home page; they were also printed in the user manual. Discussion of suicide, self harm and other traumatic topics was not permitted. To preserve their anonymity, participants were asked to employ a pseudonym and to avoid posting material that would identify them. In addition, links and email addresses were filtered out of posts and signatures using an automated script.

#### ISG+ITP: WellBeing Board plus e-couch

This condition involved the simultaneous delivery of e-couch (ITP) and a dedicated WellBeing Board (ISG) over the 12 week intervention period. To prevent contamination between the ISG only and the ISG+ITP conditions, separate ISGs were established for the two conditions. The ISG for the combined condition incorporated an additional forum that enabled participants to discuss their experiences with the ITP e-couch condition.

#### IAC: HealthWatch

The control condition was purpose-built for the current study [14]. It comprised 12 online modules each of which comprised two components (i) forced-choice and open-ended questions; and (ii) health information. The first component comprised a series of questions on a topic which was potentially related to depression and wellbeing such as nutrition, or social and family relationships or humour. For example, the online questions on humour (Week 11) commenced: “Some people believe that humour and laughter can prevent depression and increase the enjoyment of life. Today we would like to ask you to think about humour and whether it is important to your mood”. Examples of the accompanying questions were: “Do you enjoy telling jokes?”, “How often do you tell jokes?”, “What is the funniest joke you can remember?”, “Overall do you think your moods are affected by humour?” The second component consisted of information on topics related to wellbeing but containing minimal information about interventions for depression or stress [14]. Examples of topics included environmental health, nutrition myths and medicine in the home. Material for component (i) of the modules was modified and extended from questions employed in a 5-module telephone tracking Control condition in a previous study [16]. Material for component (ii) was modified from public domain health material published by authoritative US government health sources. Each week participants were asked to login to HealthWatch and complete the questions and read the material in the current HealthWatch module.

### Procedure

Participants who supplied their written consent for participation in the study were provided with a user ID and password via automated email. One week prior to commencement of the intervention these participants were prompted via another automated email to complete the online baseline survey. Thereafter participants in each of the four conditions received a weekly automated email informing them of the availability of their next module. A reminder email was sent 4 days after the initial email if the participant failed to login to the weekly module. Participants who still failed to log in received a telephone reminder to do so from their assigned project interviewer in the following week. The current week's module could only be accessed once all modules assigned in the preceding weeks had been completed. At the end of the trial, participants were advised by automated email to complete an online post intervention survey. Further emails were sent 6 and 12 months after the commencement of the intervention requesting that the participants complete follow-up online surveys.

### Randomisation and allocation concealment

Participants were randomised using a stratified block design procedure to the four conditions by the trial biostatistician (AM) who was not involved in the day-to-day conduct of the trial. Stratification variables were level of psychological distress (high/low distress level on K10), gender (male/female, unspecified), age, and location of residence (metropolitan/rural). Randomisation sequences with a fixed block size of four were generated for each strata using the Randomisation.com website (http://www.randomization.com).

### Measures

The primary outcome measure was the Centre for Epidemiologic Studies Depression scale (CES-D [17]), a 20-item self-report scale used to measure change in the severity of *depressive symptoms*. The CES-D has previously been demonstrated to have good psychometric properties [17], [18]. In the current study, the internal reliability of the CES-D at baseline was 0.74 (Cronbach's alpha) and the 14 week test-retest reliability in the Control group (baseline to post intervention) was 0.59 (Pearson correlation). *Demographic* information was collected in the screening survey or the baseline survey including gender, age, marital status, level of education completed, and employment status. Measures of self-reported *clinical characteristics* including current and past history of depression, help seeking for depression and disability associated with depression in the past month were also collected. In addition, items were included to measure participant *perceived credibility of website interventions* and participant *randomisation preference* at baseline. Finally, a series of secondary outcome measures were collected. Details of the latter, which are not the subject of the current paper, are available in the previously published description of the trial protocol [14].

### Planned sample size

Based on previous studies [16], [19] it was estimated that pre–post effect sizes for the ITP, IAC and ISG conditions would be .6, .1, and .35 respectively. Conservatively assuming a correlation of .5 between pre- and post-test measurements, a sample size of 500 (125 per group) was required to detect with 80% power a change from baseline of approximately .25 standard deviations *a priori* contrasts of treatment arms. Although well powered to detect anticipated effects of each intervention alone, it was acknowledged that the additional benefits of an ISG when added to ITP might be small and that the trial would be exploratory rather than definitive in that regard.

### Analyses

Since the data contained a number of outliers (primarily involving large and uncharacteristic changes from occasion to occasion), all CES-D data were first dichotomised into caseness (where, consistent with usual practice, a case was classified as a CES-D score>16). This outcome was analysed on an intent-to-treat basis using a mixed effects logistic model with factors of intervention arm and occasion of measurement and a random participant intercept using gllamm in Stata 10.1. Planned contrasts of model parameters tested hypotheses regarding differences in odds of change over time between interventions.

Associations between outcomes and demographic variables and dropout were investigated using t-tests and chi-square tests. ANOVAs, t-tests, Pearson chi-square, and Fisher Exact Probability tests were employed to identify any differences between conditions in baseline attributes or scores and also to compare attrition across the conditions in order to investigate attrition bias and potential threats to internal study validity.

The number needed to treat (NNT) was calculated for participants above caseness criteria at baseline with confidence intervals estimated using the method proposed by Bender [20].

## Results

### Flow of participants

**Figure 1:**
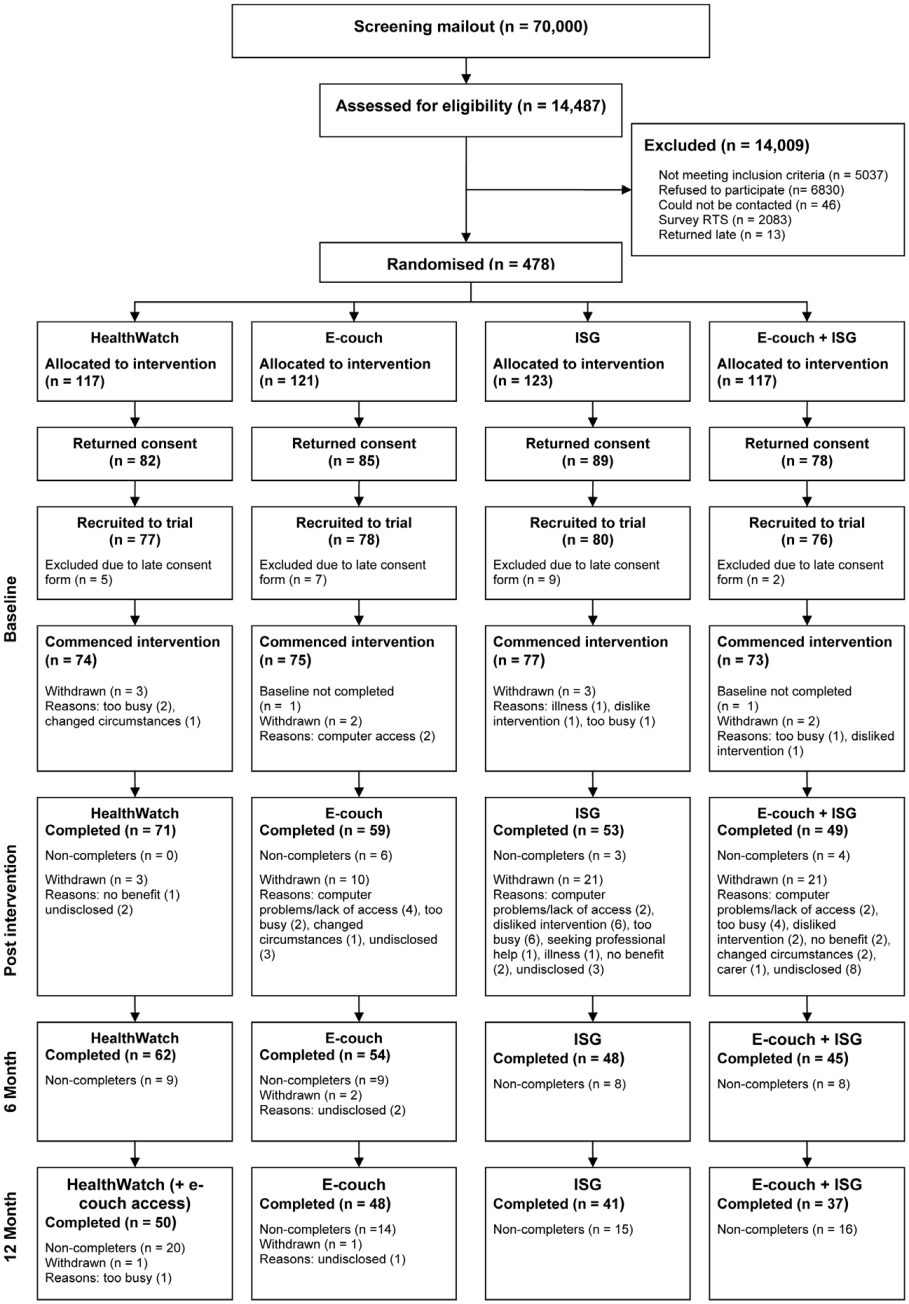
Participant flow through the study.

The participant flow for the trial is shown in Figure 1. A total of 12,391 participants returned completed screening surveys on time (18.2% response rate excluding late responders and return to senders from the denominator). Of these respondents the majority either indicated in their screening survey that they did not wish to participate in the trial (55.1%) or failed to meet one of the other eligibility criteria (40.7%). A further 46 eligible respondents could not be contacted leaving a total of 478 participants who were randomised to one of the four conditions. Of these potential participants, 167 (34.9%) either failed to return the consent form or returned it after the cut-off date for their inclusion in the intervention. The remaining 311 participants were advised of the condition to which they had been allocated and were asked to complete the online baseline survey. Twelve consenting participants (3 per condition) either formally withdrew from the study before undertaking the baseline survey or failed to submit a baseline or subsequent survey (n = 2). Of these 12 participants, one participant in the ISG condition and one participant in the ISG+ITP combined condition indicated that their withdrawal was due to the condition to which they had been allocated. Further, one participant in the e-couch condition did not complete sufficient items to compute a baseline CES-D score and provided no further CES-D data. In the absence of this data this participant could not be included in the CES-D analysis. Accordingly, the primary outcome measure analyses and the comparisons of baseline characteristics across groups were based on the 298 participants who provided sufficient data to calculate at least one CES-D score over the course of the trial (IAC n = 74, ITP n = 74, ISG n = 77, ISG+ITP n = 73).

### Baseline characteristics of participants as a function of condition

Table 1 summarises the characteristics of participants who provided at least one CES-D score in each condition.

**Table 1:** Characteristics of participants in each condition at baseline.*

	Control (n = 74)	ITP (n = 74 )**	ISG (n = 77)	ITP+ISG (n = 73 )	
Gender - men:	28 (39.2)	18 (24.3)	30 (39.0)	20 (27.4)	χ^2^(3) = 6.10, p = .11
Age (M (sd) years):	44.7 (11.34) (n = 72)	41.2 (13.07) (n = 71)	44.4 (12.4)	45.3 (11.29)	F(3, 289) = 1.69, p = .17
Completed Tertiary education	21 (28.8) (n = 73)	29 (39.2)	28 (36.8) (n = 76)	30 (41.7) (n = 72)	χ^2^(3) = 2.94, p = .40
Rural resident	38 (54.3) (n = 70)	37 (52.9) (n = 70)	35 (47.9) (n = 73)	38 (53.5) (n = 71)	χ^2^(3) = 0.71, p = .87
Employed (PT/FT)	60 (81.1)	57 (78.1) (n = 73)	58 (75.3)	52 (72.2) (n = 72)	χ^2^(3) = 1.76, p = .62
Marital status (Married/defacto)	41 (55.4)	39 (52.7)	50 (64.9)	43 (58.9)	χ^2^(3) = 2.60, p = .46
K10 (M(sd))	31.6 (4.92)	32.1 (4.24)	31.9 (5.12)	32.3 (4.64)	F(3, 294) = .31, p = .82
CES-D (M(sd))	25.93 (11.43) (n = 73)	24.3 (10.18) (n = 72)	26.2 (11.55) (n = 76)	24.4 (10.44)	F(3, 290) = .64, p = .59
CES-D>16	58 (79.5) (n = 73)	57 (79.2) (n = 72)	63 (82.9) (n = 76)	56 (76.7)	χ^2^(3) = 0.89, p = .83
Self-reported current depression	53 (71.6)	48 (64.9)	59 (76.6)	44 (61.1) (n = 72)	χ^2^(3) = 6.86, p = .33
Self reported current depression and sought help	33 (62.3) (n = 53)	27 (56.3) (n = 48)	37 (62.7) (n = 59)	30 (68.2) (n = 44)	χ^2^(3) = 1.23, p = .75
Self-reported history of depression:	62 (83.8)	66 (90.4) (n = 73)	71 (92.2)	60 (83.3) (n = 72)	χ^2^(3) = 6.86, p = .33
Some days missed or lower productivity due to depression	34 (50.7) (n = 67)	36 (55.4) (n = 65)	47 (66.2) (n = 71)	35 (56.5) (n = 62)	χ^2^(3) = 3.59, p = .31
Confident website prevent depression	38 (51.4)	40 (54.1)	37 (48.1)	29 (39.7)	χ^2^(3) = 3.41, p = .33
Confident website help understanding of depression	62 (83.8%)	61 (82.4)	68 (88.3)	58 (79.5)	χ^2^(3) = 2.23, p = .53
‘Preferred’ website†	n = 72	n = 70	n = 70	n = 69	
ITP	45 (62.5)	43 (61.4)	49 (70.0)	43 (62.3)	χ^2^(3) = 1.45, p = .69
ISG	21 (29.2)	20 (28.6)	23 (32.9)	21 (30.4)	χ^2^(3) = 0.36, p = .95
ITP+ISG	34 (47.2)	35 (50)	30 (42.9)	36 (52.2)	χ^2^(3) = 1.35, p = .72
Control	33 (45.8)	42 (60.0)	37 (52.9)	34 (49.3)	χ^2^(3) = 3.12, p = .37

Values are numbers (percentages) unless otherwise stated.

M = Mean. sd = standard deviation. CES-D Center for Epidemiological Studies – Depression. K10 = Kessler Psychological Distress Scale. ITP – Internet training program (e-couch); ISG – Internet support group (WellBeing Board); ITP+ISG –Training program and support group combined; Control (HealthWatch).

* Excludes the participants who dropped out prior to baseline (n = 12) and the participant who did not provide sufficient CES-D data to compute at least one CES-D score (n = 1).

† Participants were permitted to endorse more than one website; therefore percentages do not sum to 100.

Demographic, clinical status and symptom scores were similar across conditions. The mean age of participants across conditions was 43.9 (sd = 12.1) years, and consistent with the relative prevalence of depression in men and women, 67.4% (n = 201) of the participants were women. Just over one-third (n = 108, 36.6%) of the respondents had completed at least an undergraduate tertiary degree, approximately half (n = 148, 52.1%) were from a rural region, the majority were employed (n = 227; 76.7%) and more than half were married or in a de-facto relationship (n = 173, 58.1%).

Overall, 87.9% (n = 259) of the participants self-reported a history of depression, 68.7% (n = 204) indicated that they were currently suffering from depression and of these 62.3% (n = 127) indicated that they had sought help for depression. The mean CES-D score for the participants fell at the upper end of the mild range for depression (mean 25.2, sd = 10.91) with 79.6% (n = 234) of the participants scoring above the cutoff for clinically significant symptoms. Approximately one-quarter (25.6%, n = 65) had experienced time in the past 30 days when they were totally unable to work due to depression, over half (55.3%, n = 146) experienced periods where they could work but at a reduced level of productivity due to depression and a similar number experienced at least one of these problems (57.4%, n = 152).

Although the pattern of pre-trial preferences for the four interventions was similar across conditions, the distribution of interest in the four websites differed with 180 (64.1%), 146 (52%), 135 (48%) and 85 (30.2%) participants overall indicating an interest in the ITP, Control, ISG+ITP combined, and ISG conditions respectively.

### Dropout across conditions and characteristics of completers and non-completers

Of the 311 participants who provided consent, 17 (5.5%), 85 (27.3%), 109 (39%), and 141 (45.3%) at baseline, post-test, 6 months and 12 months respectively either failed to complete the survey or did not provide sufficient data to calculate a CES-D score (non-completers). At post-test, attrition was significantly lower in the Control group than each of the intervention conditions and at 6 and 12 months completion rates remained significantly lower in the control group than the two conditions involving an Internet Support group (see Table 2). However, the differential attrition rate was due to dropout over the intervention and immediate post-intervention phase of the trial, a series of two sample t-tests for percentages revealing no significant differences between conditions in attrition between post-test and 12-month follow-up (p>.05 in each case).

**Table 2:** Number (%) of CES-D completers (C) and non-completers (NC) for each condition at each follow-up among those providing consent for participation (n = 311).

Time of assessment	Control (n = 77)		ITP (n = 78)		ISG (n = 80)		ITP+ISG (n = 76)		
	C	NC	C	NC	C	NC	C	NC	
*Number (%):*									
Baseline	73^a^ (94.8%)	4	72^a^ 92.3%	6	76^a^ (95.0%)	4	73^a^ (96.1%)	3	χ^2^(3) = 1.13, p = .77
Post	71^a^ (92,2%)	6	58^b^ (74.4%)	20	52^b^ (65%)	28	47^b^ (61.8%)	29	χ^2^(3) = 22.82, p<.001
6 months	59^a^ (80.8%)	18	53^ab^ (67.9%)	25	47^b^ (58.8%)	33	45^b^ (59.2%)	31	χ^2^(3) = 8.61, p = .04
12 months	51^a^ (66.2%)	26	46^ab^ (59.0%)	32	39^b^ (48.8%)	41	36^b^ (47.4%)	40	χ^2^(3) = 8.68, p<.03

Note: There was no statistical difference in dropout rates for the conditions which share the same subscript.

Most baseline scores and clinical and demographic characteristics were similar for CES-D completers and non-completers. In particular, the baseline CES-D scores were not significantly different for completers and non-completers in any group at any time point except that the baseline CES-D was lower among 12 month completers in the Control condition. Similarly, in most cases, K10 scores did not differ significantly for completers and non-completers except that ITP participants who completed the CES-D at 12 months had a higher baseline K10 than non-completers and the reverse was true for ITP+ISG participants; those completing the CES-D at 12 months had a lower baseline K10 level than non-completers. There were no significant differences between completers and non-completers for caseness (CES-D>16), self-reported history of depression or current levels of depression or for whether they had experienced disability due to depression (unable to work or lowered productivity last 30 days). However, ITP CES-D completers at post, 6 months and 12 months were more likely to indicate at baseline that they had sought help for depression and a similar pattern was obtained for ISG CES-D completers at post and 6 months. The majority of demographic comparisons demonstrated no difference in dropout. In particular, there were no significant differences between the age of completers and non-completers or their location of residence (rural versus metropolitan). However, men were more likely to drop out of the ITP condition at each assessment point and out of the ISG condition at 12 months. Those with a tertiary education were less likely to drop out of the ITP condition (trend at post; 6 months, 12 months). Finally, for the ISG condition participants who completed the CES-D at 6 months were more likely to be unemployed (a similar trend was observed at 12 months, p = .064) and the Control condition completers at 6 months were more likely to be married than non-completers. There were no other gender, educational, employment or marital status differences between completers and non-completers.

### Adherence to the intervention

The adherence data for the 298 participants who provided at least one baseline CES-D score are summarised in Table 3.

**Table 3:** Intervention adherence for the participants who provided at least one CES-D measure (n = 298).

	Control (n = 74)	ITP (n = 74)	ISG (n = 77)	ITP+ISG (n = 73)
Mean (sd) weeks logged on during 12 wk intervention period	11.51 (1.62)	10.15 (3.37)	6.18 (4.18)	8.45 (4.29)
Mean (sd) *modules* completed during 12 wk intervention period	11.22 (1.72)	9.97 (3.48)	N/A	7.97 (4.56)
Mean (sd) *modules* completed during trial	11.22 (1.72)*	10.09 (3.46)	N/A	8.00 (4.57)
Number (%) completing all 12 *modules* during 12 wk intervention period	43 (58.1)%)	48 (64.9%)	N/A	32 (43.8%)
Number (%) completing all 12 *modules* during trial	43 (58.1%)*	50 (67.6%)	N/A	33 (45.2%)
Mean (sd) weeks at least 4 posts	N/A	N/A	1.96 (3.01)	.73 (1.52)
Number (%) completing ≥4 posts all 12 wks	N/A	N/A	1 (1.3%)	0 (0%)
Mean (sd) weeks at least 1 post	N/A	N/A	6.08 (4.28)	4.03 (3.83)
Mean (sd) posts over 12 wks	N/A	N/A	30.34 (83.53) Mdn = 13	9.05 (10.64) Mdn = 5
Mean (sd) posts after intervention (wks 13–24)	N/A	N/A	15.64 (88.65)	.04 (.26)
Number (%) posting (≥1 post) after intervention phase	N/A	N/A	20 (26%)	2 (2.7%)

* Excludes data from delayed access to e-couch.

#### Logons

On average, over the initial, structured, 12 week-intervention period, participants from the control, ITP, ITP+ISG and ISG conditions logged-in on 96%, 83%, 70% and 52% of weeks respectively. ITP and Control participants logged-in on significantly more weeks in the intervention period than participants in the conditions incorporating an ISG (Control vs ISG: difference = 5.33, 95% CI = 2.30–6.43, p<.001; ITP vs ISG: difference = 3.06, 95% CI = 1.99–4.21, p<.001; ITP vs ITP+ISG: difference = 1.36, 95% CI = 0.22–2.51, p = .02).

#### Module completion

During the 12 week intervention period, the control and ITP participants completed the majority of the automated modules (on average, 93.5% and 84.6% respectively). The participants in the ITP+ISG group completed two-thirds of the ITP modules during the 12 week intervention period, significantly fewer than were completed by the ITP alone or the Control group (ITP vs ITP+ISG difference = 2.00, 95% CI = 0.88–3.12, p = .001); Control vs ITP+ISG difference = 3.24, 95% CI = 2.12–4.37, p<.001). In addition, the ITP group completed significantly fewer modules than the Control group (difference = 1.24, 95% CI = 0.13–2.36, p = .029). Sixty-five percent of the ITP-alone group completed all 12 modules in the first 12 weeks of the trial compared with less than half of the ITP+ISG group, a difference which was statistically significant (χ^2^(1) = 6.55, p = .01). In addition, three ITP participants and one participant from the ITP+ISG group completed at least one additional module following the 12 week intervention phase during which period two of the ITP participants and the ITP+ISG participant finished all 12 modules.

#### Posts on ISG

Participants in the ISG conditions were asked to make 4 posts per week to the Board. Few contributed this many posts in a week and only one participant (ISG alone group) did so in each of the 12 weeks. However, on average the ISG alone participants made at least one post on each of 50% of the weeks in the intervention period; the ISG+ITP participants made at least one post on a third of the intervention period weeks. Participants in the ISG+ITP group made significantly fewer posts and logged-on in significantly fewer weeks during the intervention phase than those in the ISG alone condition (Total posts: difference = 21.28, t(78.6) = 2.22, 95% CI = 2.17–40.39, p = .03; Number of weeks≥1 post: difference = 2.05, t(148) = 3.08; 95% Confidence Interval = .74–3.36, p = .002). Participants in the ISG conditions were able to continue to post to the ISG at the end of the 12 week intervention phase. A significantly greater percentage of the ISG alone group (26%) compared to the combined ISG+ITP group (2.7%) opted to post at least once to the board in the 3 months following the intervention phase (χ^2^ (1) = 16.16, p<.001).

#### E-couch access post intervention

After the 6 month follow-up survey, Control group participants were provided with the option of accessing e-couch. Nineteen of the 74 Control participants (25.7%) who provided at least one data point accessed the intervention between 1 and 5 times of whom the majority (n = 13; 68.4%) logged onto e-couch only once. The 19 participants comprised 37.3% of the 51 Control participants who provided a 12 month CES-D.

### Level of human contact

Excluding participants who withdrew and therefore did not receive telephone call reminders, participants received an average of 1.74 (sd = 2.21) telephone reminders over the 12 week period of the intervention. This included 0.86 (sd = 1.53), 2.39 (sd = 2.38), 1.48 (sd = 1.97) and 2.42 (sd = 2.55) reminders for the Control, ITP, ISG, and ITP+ISG conditions respectively.

### Depression outcomes

Table 4 summarises the observed proportion of participants at caseness levels for depression in each condition at each measurement occasion. Parameters of the mixed effects model and significance tests for depression caseness by occasion of measurement and intervention are shown in Table 5. Contrasts involving these parameters indicated that there was a significantly greater reduction in caseness between baseline and post-intervention (endpoint) for the ITP condition and for the ITP+ISG condition than for the Control condition. There was no significant difference in the pre-post-test changes for the Control and ISG groups. However, at 6 and 12 months there was a greater reduction in caseness since baseline for the ISG and ISG+ITP conditions compared to the Control group. There was no significant difference in reduction in caseness since baseline for the ITP compared to the Control condition at either 6 month or 12 month follow up.

**Table 4:** Observed proportion of cases for each intervention as a function of measurement occasion.

	Control	ITP	ISG	ITP+ISG
Baseline	0.79 (0.69–0.87) (n = 73)	0.81 (0.70–0.88) (n = 72)	0.87 (0.77–0.93) (n = 75)	0.79 (0.69–0.87) (n = 73)
Post	0.73 (0.62–0.82) (n = 71)	0.53 (0.41–0.66) (n = 58)	0.67 (0.54–0.78) (n = 52)	0.48 (0.34–0.62) (n = 46)
6 month f/up	0.71 (0.59–0.81) (n = 59)	0.57 (0.43–0.69) (n = 53)	0.55 (0.41–0.69) (n = 47)	0.49 (0.35–0.63) (n = 45)
12 month f/up	0.65 (0.51–0.76) (n = 51)	0.58 (0.43–0.71) (n = 45)	0.46 (0.32–0.61) (n = 39)	0.42 (0.27–0.58) (n = 36)

**Table 5:** Mixed effects logistic regression model parameters and significance tests for depression caseness by occasion of measurement and intervention.

	Odds Ratio	Standard Error	z	P value	95% Confidence Interval	
Occasion of Measurement						
Endpoint	0.57	0.29	−1.09	0.274	0.21	1.56
6 month f/up	0.47	0.25	−1.40	0.161	0.16	1.35
12 month f/up	0.34	0.19	−1.94	0.053	0.12	1.01
Intervention						
E-couch	1.11	0.78	0.15	0.882	0.28	4.41
Board	2.43	1.80	1.20	0.232	0.57	10.36
Ecouch+Board	1.06	0.74	0.09	0.932	0.27	4.18
Intervention *by* Occasion of Measurement						
Endpoint - E-couch	0.19	0.15	−2.15	0.031	0.04	0.86
Endpoint - Board	0.27	0.22	−1.59	0.113	0.05	1.36
Endpoint - Ecouch+Board	0.12	0.10	−2.61	0.009	0.02	0.59
6 m follow-up - E-couch	0.32	0.25	−1.45	0.146	0.07	1.49
6 m follow-up - Board	0.11	0.10	−2.54	0.011	0.02	0.60
6 m follow-up - Ecouch+Board	0.17	0.14	−2.13	0.033	0.03	0.87
12month follow-up - E-couch	0.52	0.43	−0.79	0.431	0.10	2.62
12 m follow-up - Board	0.08	0.08	−2.73	0.006	0.01	0.49
12 m follow-up - Ecouch+Board	0.17	0.15	−2.01	0.044	0.03	0.96
Variance of random intercept: 6.02 (s.e: 1.37, ICC: 0.65)						

Notes: Reference conditions were Baseline occasion of measurement and HealthWatch intervention.

Table 6 shows the number of persons meeting criteria for depression needing to be treated for one person to be subthreshold at the endpoint of the intervention and at 6 and 12 month follow-ups. While the values of these indices generally followed the pattern of the intention to treat analyses, they were based on the smaller group of persons meeting threshold at baseline and, accordingly, power to declare an NNT index as significant was lower. Thus, although the NNT for the ISG alone condition appeared to decline across measurement occasions, falling to 5.4 at 12 months, the indices failed to achieve significance. The remaining findings were consistent with the intention to treat analyses. ITP alone had a low and statistically significant NNT of 5.05 at the end of the intervention but effectiveness was lost at follow-up. The combination of interventions resulted in low, stable and statistically significant NNT indices over all occasions of measurement.

**Table 6:** Numbers needed to benefit from treatment in participants who met criteria for diagnosis at baseline (95% CI)†.

	ITP	ISG	ITP+ISG
Endpoint	5.05 (2.84–31.73)	16.86 (4.51–∞–−10.05)	3.94 (2.40–13.52)
6 month f/up	6.86 (3.20–∞–−32.21)	6.30 (3.0190–∞–−41.51)	4.49 (2.54–29.37)
12 month f/up	14.43 (3.92–∞–−8.23)	5.39 (2.69–∞–−50.48)	4.34 (2.40–53.73)

† Confidence intervals may include infinity and extend to numbers needed to be harmed, which are represented by negative values.

## Discussion

The current study raises the possibility that over the long term an online peer-to-peer support group may be an effective intervention for reducing depressive symptoms among members of the community with elevated depressive symptoms and a self-reported history of depression. There was no change in depressive symptoms relative to the control condition after 3 months exposure to the ISG. However, both the Internet support group alone and the Internet support group combined with an automated Internet depression training program did show a significantly greater reduction in depressive symptoms at 6 and 12 months follow-up. The training program alone was effective relative to control at post-test but the effect was not significant at 6 months. Adding an ISG reduced rather than improved adherence to the training program. The Control group was provided with the opportunity to access the automated depression training program after the 6 month follow-up; at 12 months there was no significant difference between this group and the group which received the training program from the outset.

### Strengths and weaknesses of the study

To our knowledge, this is the first randomised controlled trial of the effectiveness of an online peer-to-peer support group for reducing depressive symptoms and the first to compare the effect of such a group with the effectiveness of an Internet-based automated psychotherapy intervention for depression. The intervention was associated with minimal human guidance with reminders to undertake the program being primarily delivered by automated email. Further, the study employed a credible Internet-based control condition which was associated with a high level of adherence. However, there were limitations. Uptake of the trial invitation was lower than anticipated and long term attrition was high. Given the high costs of the recruitment technique employed, it was not feasible to recruit the intended sample size. In addition, as has been frequently documented in other studies [21], [22], dropout and lack of adherence during the intervention and immediate post-intervention phase of the trial was higher among the intervention groups than for the Control group and higher in the combined intervention group than in either groups alone. It is possible that the effort required to participate in these interventions varies and with it the level of adherence and attrition. Further, some participants either withdrew prior to baseline or failed to complete the CES-D measure thus providing no data to be incorporated into the planned intent to treat analysis. In addition, the presence of outliers, including large between occasion changes, in the data necessitated the dichotomisation of the primary outcome measure. Finally, although the baseline characteristics of the completers and non-completers were broadly similar, including for the outcome measure, dropout from the training program was greater among some participants (men, those with less education, those who had previously sought help) which may affected the generalisability of the findings.

### Strengths and weakness in relation to other studies

The findings of this RCT are consistent with evidence from a previous quantitative depression ISG study reported by Houston and his collaborators [23]. In a 12-month follow up study, they found that members of a public depression ISG who participated frequently in the online group achieved a significantly greater reduction in depressive symptoms than those who infrequently posted after controlling for initial severity of depression. However, the design used in the latter study had the potential to yield biased outcomes since participants self-allocated to the low and high frequency groups. In contrast, the current study employed a randomised controlled trial methodology including a purpose constructed plausible, web-based attention control. To our knowledge, the only other directly relevant quantitative evaluation of the effect of a depression ISG, involved the *Control*-arm of an RCT of a CBT-based Internet intervention for depression [19]. The Control group activity comprised participation in a research ISG for approximately 3 months. As in the current study, there was no significant reduction in depressive symptoms among ISG participants immediately after participation in the ISG. However, the longer term effect of ISG use could not be evaluated in this study because Control participants were given access to the online CBT intervention after 3 months.

### Implications

While inviting replication, the findings of the current study have substantial practical implications for consumers, clinicians and policy makers. They suggest that ISGs may be of benefit for people with a history of depression and with current depressive symptomatology. Thus, clinicians might consider referring interested clients to such groups and policy makers have a stronger basis for funding and promoting depression ISGs. The findings further suggest that there may be value in combining standard Internet training programs with an Internet support group since the effect of the ITP occurred immediately post-intervention and tapered off and the effect of the ISG was delayed and increased over time.

### Future research

Further research is required to confirm the outcomes, to determine who might benefit most from an ISG and to identify methods for increasing participation in ISGs. There is also a need to investigate the reliability and nature of the delayed improvement effect. Participants did not log onto the ISG boards frequently following the intervention. Thus, the mechanism underlying the delayed improvement in depressive symptoms is unclear. It is possible that participants developed communication skills that they then transferred to and practised in face-to-face situations which in turn resulted in a delayed reduction in depressive symptoms. It is also possible that participation in the ISG prompted subsequent formal help seeking. Alternatively, frequency of logins may not be the only or most important indicator of participant use of the board. For example, it is possible that simply reading posts is beneficial for this group. Finally, the current study employed an ISG founded and developed for the specific purposes of the project. The culture, mechanisms of action and effectiveness of well established public ISGs may differ from that of a research ISG. Such public depression ISGs are the most widely employed and accessible services for consumers. Accordingly, we are currently undertaking a randomised controlled trial to investigate the effectiveness of an ecologically valid, established depression ISG.

## Supporting Information

Checklist S1 CONSORT Checklist
(DOC)

Protocol S1 Trial Protocol
(PDF)

## References

[1] Fox S (2009) The Social Life of Health Information. Washington DC: Pew Internet and American Life Project.

[2] Malhi G, Adams D, Porter R, Wignall A, Lape L, et al (2009) Clinical overview: Clinical practice recommendations for depression. Acta Psychiatrica Scandinavica 119 Suppl. 439: 8–26. 10.1111/j.1600-0447.2009.01382.x 19356154

[3] World Health Organization (2008) The global burden of disease: 2004 update. Geneva: World Health Organization.

[4] Berger M, Wagner TH, Baker LC (2005) Internet use and stigmatized illness. Social Science and Medicine 61: 1821–1827. 16029778 10.1016/j.socscimed.2005.03.025

[5] Griffiths KM, Farrer L, Christensen H (2010) The efficacy of internet interventions for depression and anxiety disorders: a review of randomised controlled trials. MJA 192: S4–S11. 20528707 10.5694/j.1326-5377.2010.tb03685.x

[6] Andrews G, Cuijpers P, Craske MG, McEvoy P, Titov N (2010) Computer Therapy for the Anxiety and Depressive Disorders Is Effective, Acceptable and Practical Health Care: A Meta-Analysis. PLoS ONE 5: e13196. 20967242 10.1371/journal.pone.0013196 PMC2954140

[7] National Institute for Health and Clinical Excellence (2004) Depression. The treatment and management of depression in adults. London: National Institute for Health and Clinical Excellence

[8] Horrigan JB, Rainie L, Fox S (2001) Online Communities: Networks that nurture long-distance relationships and local ties. Washington: Pew Internet & American Life Project.

[9] Davison K (2000) Who talks? The social psychology of illness support groups. American Psychologist 55: 205–217. 10717968

[10] Griffiths KM, Calear AL, Banfield M, Tam A (2009) Systematic review on Internet Support Groups (ISGs) and depression (2): What is known about depression ISGs? J Med Internet Res 11: e41. 19793718 10.2196/jmir.1303 PMC2802257

[11] Pfeiffer P, Heisler M, Piette J, Rogers M, Valenstein M (2011) Efficacy of peer support interventions for depression: a meta-analysis. General Hospital Psychiatry 33: 29–36. 21353125 10.1016/j.genhosppsych.2010.10.002 PMC3052992

[12] Eysenbach G (2005) The law of attrition. J Med Internet Res 7: e11. 15829473 10.2196/jmir.7.1.e11 PMC1550631

[13] DiMatteo MR (2004) Social support and patient adherence to medical treatment: a meta-analysis. Health Psychology 23: 207–218. 15008666 10.1037/0278-6133.23.2.207

[14] Griffiths K, Crisp D, Christensen H, Mackinnon A, Bennett K (2010) The ANU WellBeing study: a protocol for a quasi-factorial randomised controlled trial of the effectiveness of an Internet support group and an automated Internet intervention for depression. BMC Psychiatry 10: 20. 20211025 10.1186/1471-244X-10-20 PMC2850878

[15] Kessler R, Andrews G, Colpe L, Hiripi E, Mroczek, et al (2002) Short screening scales to monitor population prevalence and trends in nonspecific psychological distress. Psychological Medicine 32. 10.1017/s0033291702006074 12214795

[16] Christensen H, Griffiths K, Jorm A (2004) Delivering interventions for depression by using the internet: Randomised controlled trial. British Medical Journal 328: 265–270. 14742346 10.1136/bmj.37945.566632.EE PMC324455

[17] Radloff LS (1977) The CES-D scale: A self report depression scale for research in the general population. Appl Psych Meas 1: 385–401.

[18] Eaton W, Muntaner C, Smith C, Tien A, Ybarra M (2004) Center for Epidemiologic Studies Depression Scale: Review and Revision (CESD and CESD-R). In: Maruish M, editor. The Use of Psychological Testing for Treatment Planning and Outcomes Assessment. Third ed. Mahwah, NJ: Lawrence Erlbaum Associates. pp. 363–377.

[19] Andersson G, Bergstrom J, Hollandare F, Carlbring P, Kaldo V, et al (2005) Internet-based self-help for depression: Randomised controlled trial. British Journal of Psychiatry 187: 456–461. 16260822 10.1192/bjp.187.5.456

[20] Bender R (2001) Calculating confidence intervals for the number needed to treat. Controlled Clinical Trials 21: 102–110. 10.1016/s0197-2456(00)00134-3 11306148

[21] Christensen H, Griffiths KM, Farrer L (2009) Adherence in Internet interventions for anxiety and depression: Systematic review. Journal of Medical Internet Research 11 2: e13. 19403466 10.2196/jmir.1194 PMC2762797

[22] Lintvedt OK, Griffiths KM, Sørensen K, Østvik AR, Wang CEA, et al (2011) Evaluating the effectiveness and efficacy of unguided internet-based self-help intervention for the prevention of depression: a randomized controlled trial. Clinical Psychology & Psychotherapy Epub ahead of print 10.1002/cpp.770 21887811

[23] Houston TK, Cooper LA, Ford DE (2002) Internet support groups for depression: A 1-year prospective cohort study. American Journal of Psychiatry 159: 2062–2068. 12450957 10.1176/appi.ajp.159.12.2062

